# Comparative study of machine learning approaches integrated with genetic algorithm for IVF success prediction

**DOI:** 10.1371/journal.pone.0310829

**Published:** 2024-10-11

**Authors:** Shirin Dehghan, Reza Rabiei, Hamid Choobineh, Keivan Maghooli, Mozhdeh Nazari, Mojtaba Vahidi-Asl

**Affiliations:** 1 Department of Health Information Technology and Management, School of Allied Medical Sciences, Shahid Beheshti University of Medical Sciences, Tehran, Iran; 2 Department of Laboratory Sciences, School of Allied Medical Sciences, Tehran University of Medical Sciences, Tehran, Iran; 3 Department of Biomedical Engineering Science and Research Branch Islamic Azad University, Tehran, Iran; 4 Faculty of Computer Science and Engineering, Shahid Beheshti University, Tehran, Iran; Shiraz University of Medical Sciences, ISLAMIC REPUBLIC OF IRAN

## Abstract

**Introduction:**

IVF is a widely-used assisted reproductive technology with a consistent success rate of around 30%, and improving this rate is crucial due to emotional, financial, and health-related implications for infertile couples. This study aimed to develop a model for predicting IVF outcome by comparing five machine-learning techniques.

**Method:**

The research approached five prominent machine learning algorithms, including Random Forest, Artificial Neural Network (ANN), Support Vector Machine (SVM), Recursive Partitioning and Regression Trees (RPART), and AdaBoost, in the context of IVF success prediction. The study also incorporated GA as a feature selection method to enhance the predictive models’ robustness.

**Results:**

Findings demonstrate that AdaBoost, particularly when combined with GA feature selection, achieved the highest accuracy rate of 89.8%. Using GA, Random Forest also demonstrated strong performance, achieving an accuracy rate of 87.4%. Genetic Algorithm significantly improved the performance of all classifiers, emphasizing the importance of feature selection. Ten crucial features, including female age, AMH, endometrial thickness, sperm count, and various indicators of oocyte and embryo quality, were identified as key determinants of IVF success.

**Conclusion:**

These findings underscore the potential of machine learning and feature selection techniques to assist IVF clinicians in providing more accurate predictions, enabling tailored treatment plans for each patient. Future research and validation can further enhance the practicality and reliability of these predictive models in clinical IVF practice.

## 1. Background

### 1.1 Introduction

Confronted with the increasing prevalence of infertility, this decade has faced a sharp rise in the application of assisted reproductive technologies globally [1, 2]. In vitro fertilization (IVF), the most common and widely used procedure in ART, is a process for breeding an embryo in vitro, which, if successful, leads to pregnancy.

Since the birth of the first IVF baby in 1978 [3], efforts have been made to improve the success of IVF. However, the success rate has remained roughly constant at about 30% [4, 5] which, along with severe side effects [6, 7] and a financial burden [8, 9], make parenthood a long road for infertile couples. IVF failure can also bring emotional distress such as anxiety and stress, which may affect the quality of life and even lead to marriage failure [10, 11]. This makes "How likely can my IVF be successful?" become the most important question for infertile couples seeking treatment. Typically, to answer this question, the IVF clinicians need to consider all the demographic and clinical variables related to both female and male. With a variety of variables and their complex relations, providing an accurate estimation of success chance is challenging. Therefore, accurate models are required to predict IVF success appropriately [12, 13].

Machine learning (ML), as a subset of artificial intelligence can predict clinical outcome, by developing prediction models, based on these contributing factors. Robust prediction models can allow IVF clinicians to estimate IVF success outcome more accurately.

It is crucial to recognize that the robustness of any prediction model mainly depends on two critical factors: the choice of machine learning algorithm and the selection of the most contributing and informative features [14, 15]. Feature selection plays a pivotal role in enhancing model performance by identifying the most significant features. However, it is still a challenge to integrate the right set of features with the right ML algorithm [16, 17].

While various studies have applied machine learning techniques to develop IVF prediction models, many have relied on filter methods for feature selection [18–22], which often overlook complex interactions among variables and fail to capture the intricate relationships inherent in IVF data [23, 24]. In this study, we introduce the Genetic Algorithm as a robust wrapper method to explore the entire solution space, dynamically identifying an optimal subset of features that contribute to IVF success prediction. This approach is more flexible and effective than traditional filter methods, as it accounts for complex interactions among features.

We systematically compare the performance of five well-known machine learning algorithms—Random Forest (RF), Artificial Neural Network (ANN), Support Vector Machine (SVM), Recursive Partitioning and Regression Trees (RPART), and AdaBoost—in predicting IVF outcomes. This comparison is enhanced by the application of GA-based feature selection. By combining GA for feature selection with the aforementioned machine learning techniques, we tried to develop a robust prediction model for IVF success, offering potential improvements over existing models that rely on traditional feature selection methods.

The remainder of this paper is organized as follows: In section 1.2, we provide an overview of the previous studies, in Section 2, we describe the dataset used in this study, including the features and preprocessing steps. Section 3 describes the methodology, including the machine learning techniques applied, the Genetic Algorithm for feature selection, and the experimental setup. Section 4 presents the results of our experiments, comparing the performance of different models with and without GA-based feature selection. In Section 5, we discuss the implications of our findings, including the strengths and limitations of our approach. Finally, Section 6 concludes the paper with a summary of our contributions and suggestions for future research.

### 1.2 Related works

For the prediction of IVF outcome, the majority of the prediction models; have applied filter methods for feature extraction algorithms, but few studies have applied wrapper methods to explore their impact on the model performance. Table 1 illustrates these studies, a basis for comparison and discussion.

**Table 1:** Literature survey of ML for IVF success prediction.

Reference	Dataset	Feature selection methods	ML techniques	Results
Tian, et al. 2023 [25]	106,640 IVF/ICSI cycles	Clinical expert’s advice	Bayesian network model	Accuracy = 91.3, AUC = 0.997
Yang, et al. 2022 [18]	369 cycles	Univariate& multivariate Logistic regression analyses	Random Forest	AUC = 0.817
Wen, et al. 2022 [26]	1507 IVF/ICSI cycles	expert clinical experience	Comparing Logistic Regression, Random Forest, Support Vector Machine, Light Gradient Boosting Machine, XGBoost, Multilayer Perceptron	XGBoost with AUC = 0.787
Amini, et al. 2021 [27]	6071 cycles	principle component approach (PCA)	Comparing logistic regression, SVM/XG Boost, RF, Naïve Base/Liner Discriminant Analysis	RF with AUC = 0.60
Vogiatzi, et al. 2019 [28]	426 cycles	t-test & correlation	ANN (multilayer perceptron)	Accuracy = 74.8
Qiu et al. 2019 [29]	7188 cycles	previous studies and guidelines	Comparing Random Forest, XGBoost, Support Vector Machine, Logistic regression	XGBoost with AUC = 0.73

Tian et al. [25] aimed to predict fertilization failure in ART treatments using Bayesian network (BN) modeling. Analyzing 106,640 IVF/ICSI cycles from a Chinese reproductive health center, the study incorporated 24 predictors, including female, male, and treatment-related variables. BN modeling yielded a predictive model with 91.7% accuracy. Results showed strong calibration, with ROC AUCs of 0.779 (TFF vs. control) and 0.807 (TFF vs. LRF). Limitations include reliance on single-center data and absence of detailed IVF laboratory parameters, necessitating further validation and refinement.

Yang et al. [18] analyzed the risk factors affecting clinical pregnancy outcomes in patients undergoing in vitro fertilization embryo transfer (IVF-ET) and constructed a predictive model based on these factors. Data from 369 women undergoing IVF-ET were analyzed in this nested case-control study. Univariate and multivariate logistic regression analyses identified potential predictors, while a random forest model was validated using ten-fold cross-validation. Results highlighted age, BMI, cycle number, hematocrit, LH, progesterone, endometrial thickness, and FSH as predictors associated with clinical pregnancy outcome. Limitations included a small sample size and lack of external validation, emphasizing the need for larger-scale studies to validate these findings.

Wen et al. [26] developed an AI model for predicting pregnancy outcomes and multiple pregnancy risks using data from 1507 fresh embryo transfer cycles, encompassing 20 features,. Six machine learning algorithms were applied, with XGBoost demonstrating superior performance. The pregnancy prediction model achieved an accuracy of 0.716, and an AUC of 0.787. Limitations such as sample size constraints and exclusion of certain treatment modalities warrant further investigation.

Amini et al. [27] aimed to categorize successful IVF deliveries based on couples’ characteristics and available reproductive data using various classification methods. Conducted at a Tehran infertility center, the study collected data from 6071 IVF cycles spanning three years. Six machine learning approaches were employed, with Random Forest (RF) showing the highest accuracy (ACC = 0.81) in predicting successful delivery. Despite limitations such as single-center data and unrecorded predictors, the study highlights the importance of predictive modeling in optimizing IVF outcomes.

Vogiatzi et al. [28] developed an artificial neural network (ANN) for predicting live birth outcomes in ART using 257 infertile couples’ data from 426 IVF/ICSI cycles. Identified 12 significant parameters for ANN construction, achieving 74.8% accuracy. Limitations included the need for a more diverse cycle and infertility factor inclusion. Encouraged external validation and multicenter collaboration for enhanced applicability.

Qiu et al. [29] in their study analyzed data from 7188 women undergoing initial IVF at a Chinese medical center (2014–2018), developing machine learning models with pre-treatment variables. XGBoost demonstrated the best performance, achieving an AUC of 0.73. Limitations included the single-center nature of the study, exclusion of previously treated couples, and absence of family genetic history and lifestyle factors in the dataset.

## 2. Materials and methods

### 2.1 Dataset description

**Fig 1:**
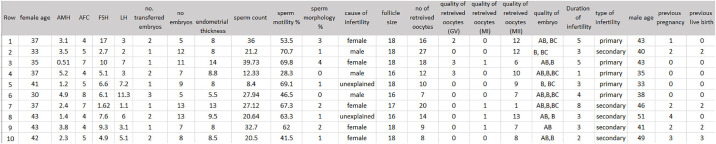
Sample image of dataset.

Medical records of couples undergoing IVF cycles at Royesh clinics, Helal-e-Iran Hospital located in Tehran, Iran, were reviewed for inclusion in this study. For each couple, only the fresh cycle of ovarian stimulation was considered. As a result, donor oocytes or embryos, frozen oocytes/embryos, and PGD/PGS cycles were excluded. A total of 812 patients met the inclusion criteria. This study was approved by the Ethics Committee of Shahid Beheshti University of Medical Sciences (IR.SBMU. RETECH.REC.1400.695). All data were de-identified and used with unique patient identifier codes. Relevant data, including demographics, medical/reproductive history of both partners, baseline information, test results, clinical diagnosis, and the treatment procedure, were extracted and recorded in a database. Fig 1 presents an image of the dataset.

All variables were extracted from textbooks, papers, and guidelines. Then an expert panel consisting eight infertility specialists was conducted. During this process, experts’ opinions toward the initial list of variables were collected based on a Likert-type scale checklist. There was an open question about whether they could add any other contributing variables that were not in the variable list. 26 variables were specified, one for treatment outcome. Table 2 shows the variables and their characteristics. The primary outcome was defined as clinical pregnancy as a positive β-HCG test result after the treatment cycle. Outcomes were obtained through a review of medical records.

**Table 2:** Descriptive statistics of variables for clinical pregnancy and non-clinical pregnancy groups.

Variable	Total N = 812 Mean (SD)	Pregnancy N = 189 Mean (SD)	Non-pregnancy N = 623 Mean (SD)
**Female Age** (Years)	37.31 (47.5)	35.60(4.62)	37.84(5.61)
**Male Age** (Years)	41.45 (5.50)	40.67(4.92)	41.69(5.65)
**Cause Of Infertility**(n,%)			
Female	252(31.03)	42(22.22)	210(33.71)
Male	108(13.30)	20(10.58)	88(14.12)
Unexplained	220(27.09)	77(40.74)	143(22.95)
**Type Of Infertility**(n,%)			
Primary	452(55.66)	111(58.73)	344(55.22)
Secondary	305(37.56)	62(32.80)	243(39)
**Duration Of Infertility**(Years)	6.17 (4.40)	4.71(3.18)	6.88(4.59)
**Smoking**(n,%)			
Yes	4(0.49)	1(0.53)	3(0.48)
No	798(98.27)	187(98.94)	611(98.07)
**Vitamin D3** (ng/ml)	33.06(15.84)	38.32(18.21)	35.50(15.94)
**Previous Pregnancy**	1.13(1.31)	0.98(1.28)	1.18(1.32)
**Previous Live Birth**	0.71(0.99)	0.68(1.01)	0.72(0.99)
**History Of Pcos**(n,%)			
Yes	115(14.16)	31(16.40)	84(13.48)
No	679(83.62)	155(82.01)	524(84.11)
**AMH**(ng/ml)	3.17(3.25)	4.31(3.40)	2.96(3.20)
**AFC**	4.87(2.66)	5.52(2.80)	4.68(2.59)
**FSH**(IU/L)	7.06(4.01)	6.94(4.11)	7.51(4.73)
**LH**(IU/L)	5.61(4.20)	6.84(5.80)	6.93(5.31)
**Endometrial Thickness**(mm)	8.60(0.74)	8.46(0.81)	8.37(0.87)
**Follicle Size** (mm)	18.67(1.53)	18.77(1.42)	18.64(1.57)
**Number Of Extracted Oocytes**	10.91(8.16)	13.38(8.15)	10.16(8.02)
**Quality Of Extracted Oocytes(MI)**	0.87(1.80)	1.16(2.20)	0.78(1.65)
**Quality Of Extracted Oocytes (MII)**	6.98(4.68)	8.48(4.41)	6.53(4.67)
**Sperm Count** (million/ml)	26.40(12.20)	26.68(12.36)	24.32(14.35)
**Sperm Motility**	55.65(17.74)	50.02(24.91)	47.14(26.47)
**Sperm Morphology**	1.65(1.09)	1.72(1.01)	1.63(1.11)
**The Number of Embryos**	6.31(4.30)	7.66(4.39)	5.89(4.19)
**The Quality of Embryos**(n,%)			
A	7(0.84)	3(1.58)	4(0.64)
AB	299(36.82)	112(59.26)	187(30.02)
B	669(82.39)	161(85.18)	508(81.54)
BC	374(46.06)	81(42.85	293(47.03)
C	66(8.13)	16(8.46)	50(8.02)
**Number Of Transferred Embryos**	1.30(0.47)	1.39(0.49)	1.28(0.46)

### 2.2 Data pre-processing

Data pre-processing is an essential part of data mining, and its goal is to get the data ready for the crucial learning model stage. The goal is to improve data quality and make it easier to understand the rules generated by the models, reducing the number of variables.

First, the collected data is divided into two groups: the target variable, which was the treatment outcome, and the predictor variables, as were the remaining ones. As the dataset includes 26 variables, 25 of them are considered predictors and one is the target class.

Among the variables, “smoking” was excluded since its values were constant in more than 90% of cases. Moreover, variables including “type of infertility” and “PCOs” were also excluded while their correlation was about zero. Finally, 22 variables remained as predictors to perform classification.

None of the variables had missing values for more than 50% of the dataset. For variables with missing values less than 50%, techniques for estimating and replacing missing values were employed. The average imputation and mode imputation methods were used to replace the missing values of numerical and categorical variables. Table 2 illustrates the distribution of the normal ranges for these features. In addition, four variables: FSH, LH, AMH, AFC, and vitamin D3, were divided into three categories (high, low, and normal) to convert discrete features into nominal values. Table 3 presents the range of these variables.

**Table 3:** Distribution of normal values.

Attribute	Normal Values
FSH	5< = FSH _(mu/ml)_< = 20
LH	5< = LH_(mu/ml)_< = 22
AMH	1< = AMH_(ng/ml)_< = 3.5
AFC	6< = AFC_(no)_< = 16
Vitamin D3	50< = D3_(ng/ml)_< = 75

### 2.3 Proposed feature selection model

Feature selection was performed exclusively using a Genetic Algorithm (GA). The GA was chosen for its ability to effectively explore the entire feature space, identifying the subset of features that contributed most significantly to classifier performance. Unlike filter methods, GA evaluates combinations of features by iteratively evolving a population of candidate solutions, thereby capturing complex interactions among features that may not be apparent through simpler methods. It aims to improve the performance and efficiency of data mining algorithms by reducing dimensionality, removing irrelevant or redundant features, and enhancing interpretability.

In this study, the feature selection process involves two stages. In the first stage, GA is employed as a feature reduction mechanism to identify discriminative features and remove redundant data. In the second stage, the best subset of features obtained by GA is used as input for different data mining techniques.

GA works with a population and generates improved solutions iteratively. Until satisfactory results are obtained, GA creates successive populations of potential solutions represented chromosomes. In the evaluation process, a fitness function assesses the quality of each solution. The crossover and mutation functions are two important operators that significantly affect the fitness value. Chromosomes for reproduction are selected based on their fitness value, with a higher probability of selection for those with greater fitness. The fittest chromosomes are more likely to be chosen for the recombination pool using either the roulette wheel or tournament selection methods. Mutation involves the random updating of genes. Crossover is a genetic operation that combines different characteristics from pairs of subsets to create a new subset. The offspring replaces the previous population using either the elitism or variety replacement strategy, resulting in a new population for the next generation. To improve performance, selected features based on genetic algorithms are used as input for classifiers.

**Fig 2:**
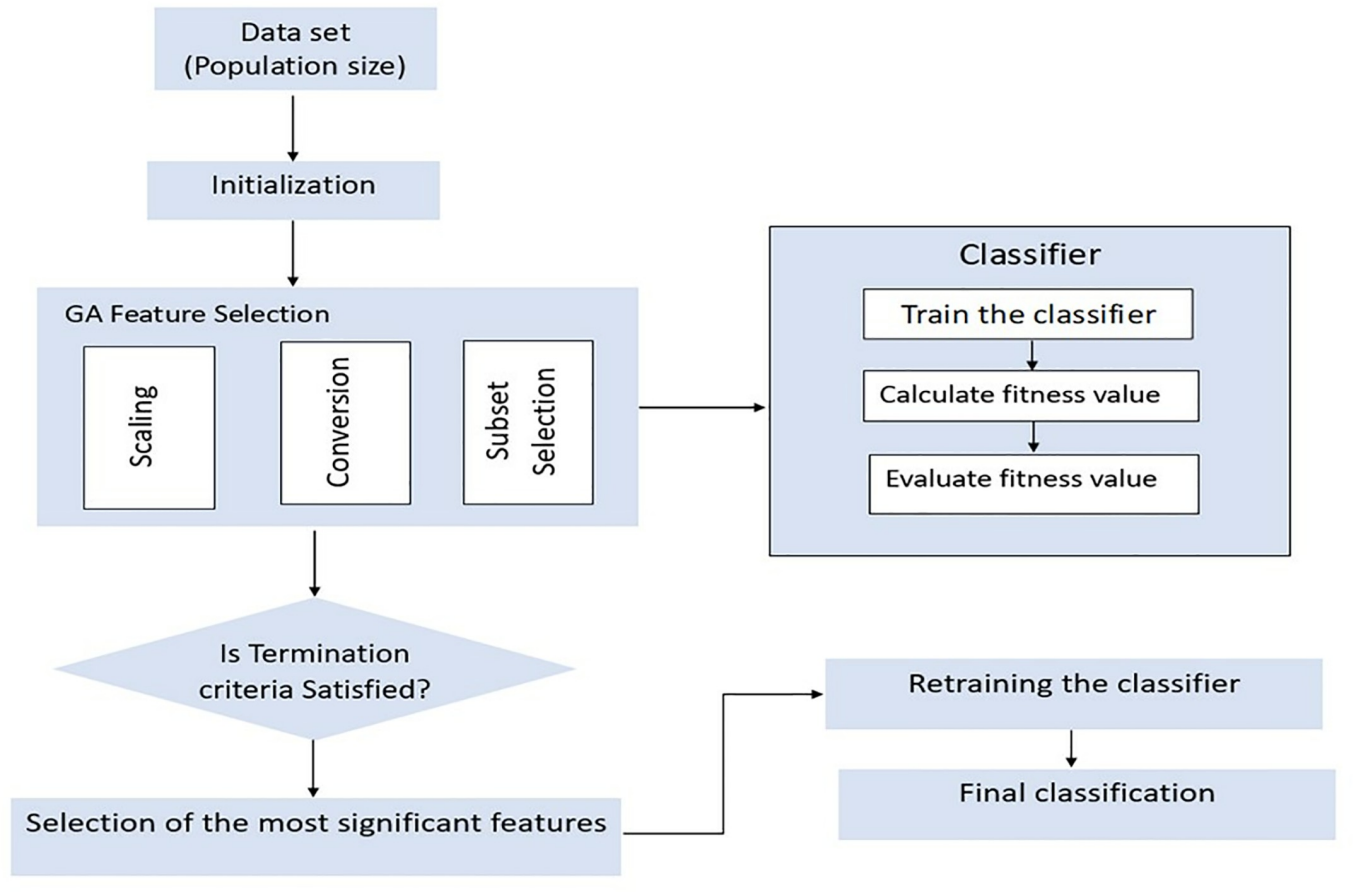
Proposed genetic algorithm for feature selection.

To model the fitness function, three criteria need to be considered: the accuracy of the model, the number of selected features, and the cost. A chromosome will have a satisfactory fitness value if it meets the acceptable classification accuracy rate, selects only significant and informative features, and reduces costs. Chromosomes with higher fitness values are more likely to be used in the next generation, as they align with user specifications. To achieve accurate feature selection using GA, the following steps should be followed. Fig 2 illustrates the application of the GA algorithm to feature selection.

- Data scaling: It offers two benefits. First, it allows controlling attributes within a smaller numeric range rather than a larger one. Second, it helps avoid numerical calculation issues.
- Conversion: The process of converting genotype to phenotype involves transforming each feature chromosome.
- Subset selection: Feature subset refers to a selected group of features chosen for analysis or modeling.
- Fitness evaluation: It is the assessment of how well a particular solution or individual performs to the desired outcome or objective.
- Termination criteria: It determines when the process should be stopped. If the criteria are met, the process ends; otherwise, it continues with the next generation.
- Genetic operation: It is a step in which genetic operations are used to search for a better solution within the GA algorithm.

### 2.4 Model training & evaluation

In this study, ANN, RPART, RF, and SVM were trained to predict pregnancy outcomes after IVF. Each of these techniques was chosen for its robustness and effectiveness in handling complex datasets. In addition, we applied an Adaboost constructed on the four mentioned classifiers and compared their performance to select the most robust prediction model.

**Fig 3:**
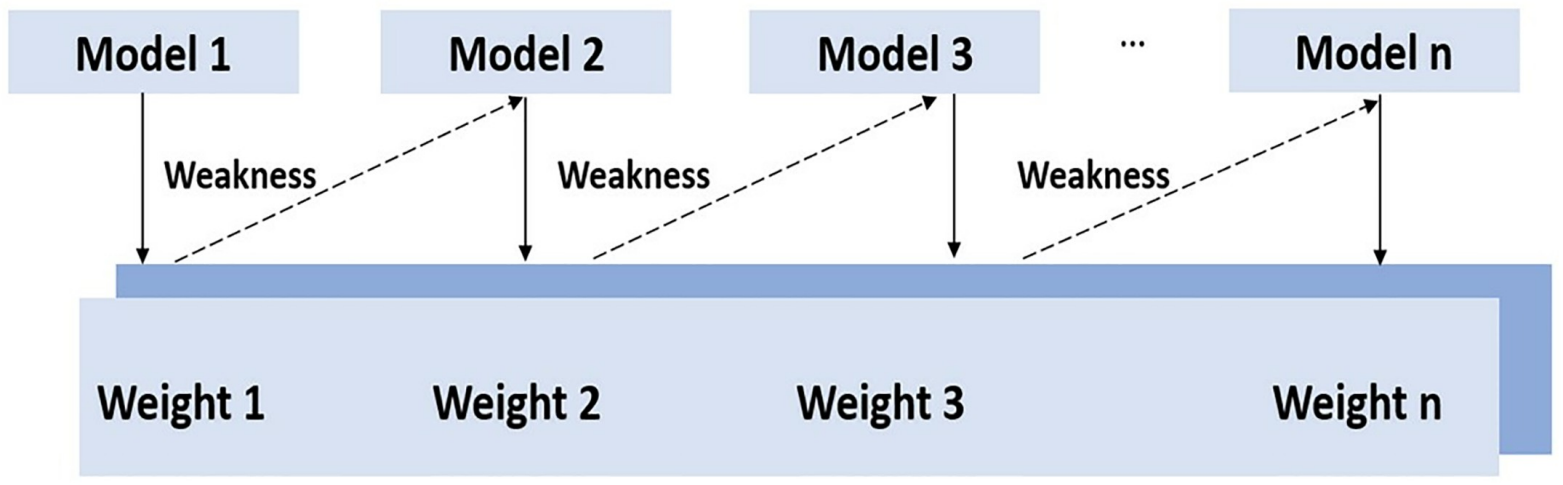
Working process of Adaboost.

- **Artificial Neural Network (ANN)**
  ANN consists of layers of neurons, which are interconnected by modifiable weights, represented by links between layers. The size of the input and output layers is determined by the number of variables in input and output data, respectively. In this study, we used 200 neurons with 100 epochs to train the model.
- **Recursive Partitioning and Regression Trees (RPART)**
  RPART is a recursive partitioning algorithm used for classification and regression in decision trees. The algorithm uses a binary tree structure to represent the decision rules and is built by recursively splitting the data into smaller subsets until a stopping criterion is met. The Gini index was used as the impurity measure and the maximum depth was determined as five.
- **Random Forest (RF)**
  The random forest method is a technique that uses many decision trees to solve classification and regression. The trees are built independently by randomly selecting vectors and have the same distribution in the forest. The error rate converges to a limit as the number of trees in the forest increases. We built the model using 1000 tress with maximum depth of three.
- **Support Vector Machine (SVM)**
  As a method for classification and regression, SVM uses a kernel that first maps the input space into a higher-dimensional feature. Then, a hyperplane is constructed in the transformed space to classify the dataset. we used a linear kernel with c = 1 in this study.
- **AdaBoost**
  AdaBoost, also known as Adaptive Boosting, is a machine learning technique used as an ensemble method. This meta-algorithm combines multiple weak classifiers and assigns equal initial weights to each sample. After each training round, the weights of the samples were adjusted based on their classification errors. The weight of misclassified samples is increased to give them more importance in subsequent iterations. Through this iterative process, k weak learners are obtained. Finally, a weighted combination is performed to obtain a strong learner.
  The working process of Adaboost is depicted in Fig 3.

During the data preparation phase, a certain number of models are selected. The first-choice model is created, and priority is given to incorrectly classified records in this initial model. Only these records were used as input for the next model. This process continues until multiple base models are determined. It’s important to note that redundant records are allowed with all the supporting methods. Algorithm 1 illustrates how the first model is created and identifies the errors made.

**Algorithm 1: Pseudocode of Adaboost**

Given (*x*_1,_ *y*_1_),…,(*x*_*n*,_ *y*_*n*_); *x*_1_ ∈ *y*_1,_ ∑*y* = {−1,1}

Initialize *D*_1_ (*i*) = 1/*n*

For t = 1 … T:

 1. Train the weak classifier using *D*_*i*_

 2. Get weak hypothesis *h*_*t*_: *X→*{−1,1}*error*

  ∑*kh*_*t*_ (*x*_*i*_) ≠ *y*_*i*_*D*_*t*_(*x*_*i*_)

 3. Choose $a_{t}=\frac{1}{2}\text{log}\left(1-E_{t}/e_{i}\right)$

 4. Update

  *D*_*t*+1_(*i*) = *Dt*(*i*)/*Z*_*t*_

 5. Output *H*(*x*) = *sign* (*∑T*_*t=1*_*a*_*t*_*H*_*t*_*(x)*)

Records that are classified incorrectly serve as input to the subsequent model. This iterative process repeats until a predefined condition is met. As can be seen, there are ’n’ numbers of models created by using the errors from the previous model. This is how the supporting process works. Models 1, 2, 3…, N represent individual classification models that can be considered as supporting models. Various supporting models operate based on the same principle.

As the dataset was highly imbalanced (189 samples in class “yes” and 623 samples in class “no”), oversampling was used. Oversampling helps mitigate this problem by generating synthetic examples for the minority class, thereby increasing its presence in the dataset. We utilized SMOTE (Synthetic Minority Over-sampling Technique) for this purpose. SMOTE generates synthetic samples by interpolating between neighboring instances of the minority class and creating new instances along the line segments. The resulting balanced dataset provides a better representation of all classes, allowing data mining methods to learn from more diverse examples and potentially improve their performance in accurately predicting the minority class. We used the Python programming language with the help of Scikit-Learn tools. Balancing was performed only on the training set. To ensure that the synthetic data generated by SMOTE is consistent with the original data, we conducted the Kolmogorov-Smirnov (KS) test to compare the distributions of the original minority class data and the synthetic samples generated by the SMOTE algorithm. By calculating the empirical Cumulative Distribution Functions (CDFs) for each feature in both datasets, we obtained the KS statistic, which measures the maximum difference between the CDFs.

Model validation was performed by random data allocation into training and test sets and by k-fold cross-validation with 10 iterations. Four measures: accuracy, precision, recall and, F-measure were used to evaluate the performance of the prediction models. In addition, we included the AUC (area under the ROC curve) metric, commonly used in medical data mining tasks. These metrics can be calculated using the following equations:

$$accuracy=\frac{number\;of\;correctly\;predicted\;samples\;}{total\;number\;of\;samples}$$

$$percision=\frac{number\;of\;correctly\;predicted\;samples\;}{number\;of\;predicted\;samples}$$

$$recall=\frac{number\;of\;correctly\;predicted\;samples\;}{number\;of\;correct\;samples}$$

$$F-Measure=\frac{2\times Percision\;\times Recall\;}{Percision\;+Recall}$$

## 3. Results

In this study, the performance of the five Machine learning techniques in terms of performance metrics was compared to each other using the proposed feature-selection method (GA). To achieve this, a dataset of 812 records with 22 variables was used. We utilized SMOTE to deal with the imbalanced data. The comparative study of obtained accuracy with and without the application of SMOTE on imbalanced datasets is provided in Table 4. As can be seen, SMOTE increased the classification accuracy of all techniques.

**Table 4:** Classifiers performance with and without using SMOTE.

Classifier	With SMOTE (accuracy%)		Without SMOTE (accuracy%)	
	With GA	Without GA	With GA	Without GA
ANN	80.7	82.1	83.2	84.5
RPART	79.6	81.3	83.4	85.1
RF	81.6	83.2	85.8	87.4
SVM	79.2	81.5	83.2	85.3
Adaboost	**83.7**	**84.7**	**87.3**	**89.8**

Table 5 summarizes the result of the KS test. As can be seen, high p-values from the KS test were obtained for all features that did not reject the null hypothesis. This suggests that there is no significant difference between the distributions. Finally, it can be concluded that the synthetic data is consistent with the original data and SMOTE effectively captured the characteristics of the original minority class, ensuring its adequacy for model training.

**Table 5:** Classifiers performance with and without using SMOTE (*Alpha = 0.05).

Features	KS Statistics	P-Value
Female age	0.017	0.992
AMH	0.036	0.072
FSH	0.007	0.083
No embryos	0.065	0.068
Endometrial thickness	0.036	0.063
Sperm count	0.004	0.097
Sperm morphology	0.008	0.083
Follicle size	0.005	0.071
No of retrieved oocytes	0.015	0.087
Quality of retrieved oocytes (MI)	0.021	0.091
Quality of retrieved oocytes (MII)	0.018	0.073
Quality of embryo	0.008	0.512

Also, Table 6 compares the performance of the five classifiers with and without the genetic algorithm as a feature selection method. For each classifier, the same training was performed but with different model parameters. 10-fold cross-validation was applied to evaluate the performance of the models on the balanced dataset.

**Table 6:** Classifiers performance with and without feature selection.

Classifier	Without GA				With GA			
	Accuracy	Precision	Recall	F1	Accuracy	Precision	Recall	F1
ANN	83.2	83.1	78.9	79.9	84.5	85.3	83.1	84.2
RPART	83.4	81.2	78.9	79.9	85.1	84.8	84.9	84.9
RF	85.8	85.5	83.1	85.3	87.4	85.7	87.8	86.7
SVM	83.2	82.4	80.9	81.4	85.3	86.0	84.4	85.2
Adaboost	**87.3**	**86.3**	**84.5**	**86.3**	**89.8**	**87.8**	**90.0**	**89.3**

The results indicate that Adaboost outperforms other classifiers regardless of whether the GA feature selection method is implemented or not. Its accuracy score was 87.3% without using the GA feature selection method, while it is further raised to 89.8% when GA is employed. Following Adaboost, RF surpassed other classifiers with an accuracy of 85.8% and 87.4% with and without the GA feature selection method, respectively. Overall, the results show a relatively significant difference in the performance of all classifiers when feature selection with GA was applied. When GA was used as the feature selection method, all classifiers achieved higher values for each performance measure.

**Fig 4:**
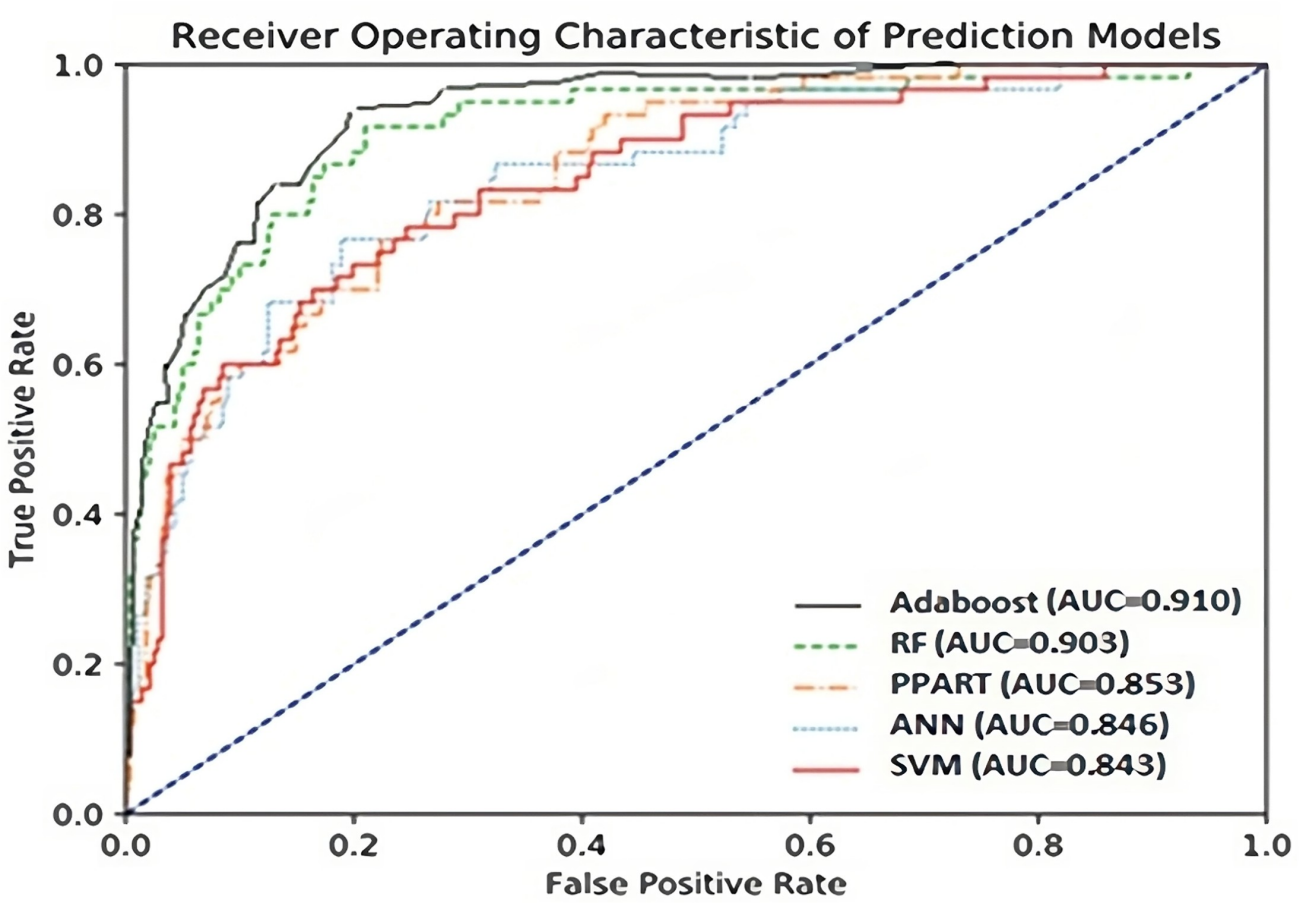
ROC curve of ANN, RPART, RF SVM, and AdaBoost models.

The predictive abilities of the classifiers were further analyzed with receiver operating characteristic (ROC) curves. As shown in Fig 4, the five classifiers showed little differences, but Adaboost had the highest AUC of 0.910. Again, RF surpassed the other classifiers, with a slight difference from Adaboost, with an AUC score of 0.903.

**Fig 5:**
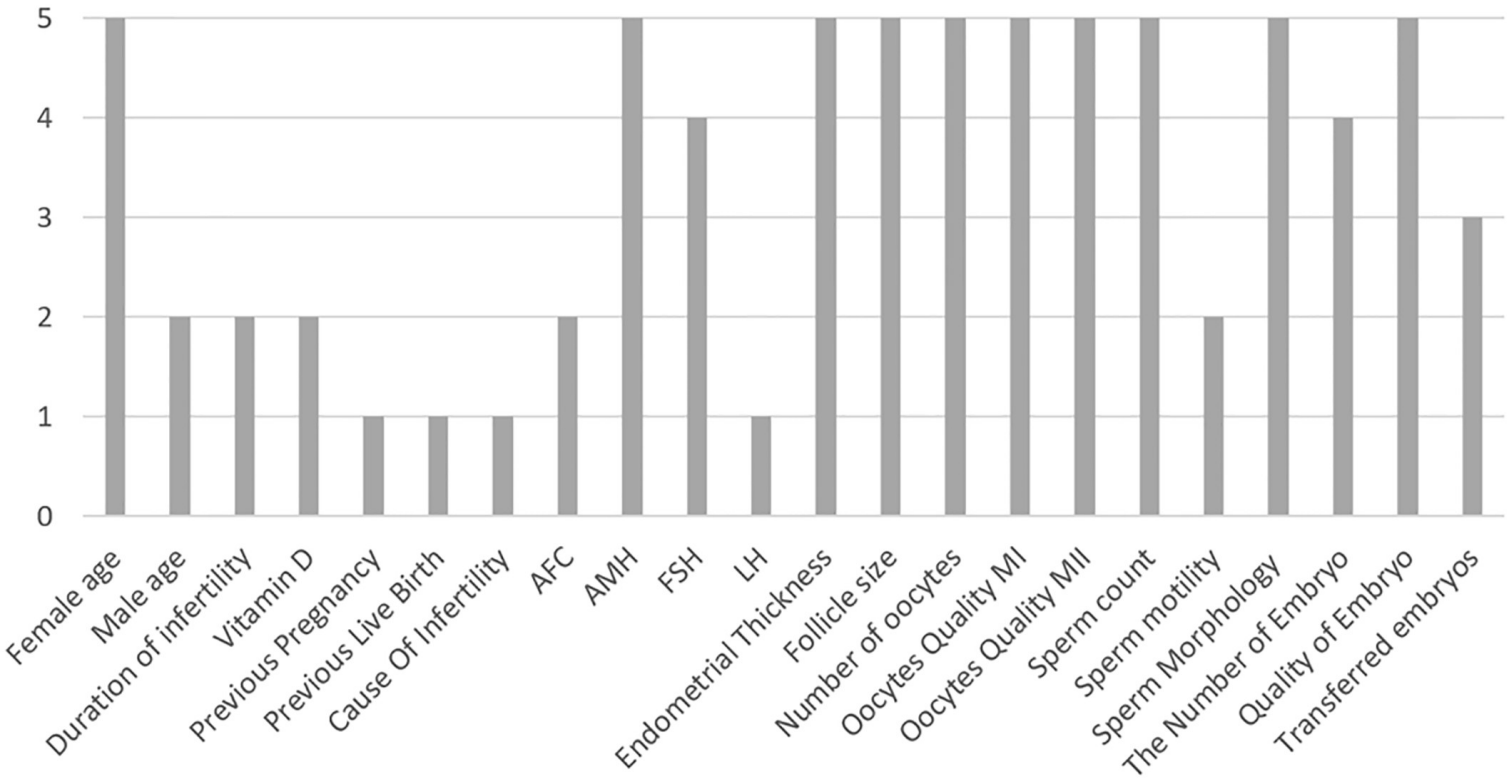
Feature subsets obtained for each classifier.

By employing each classifier as an evaluation function, the Genetic Algorithm seeks to identify the optimal feature combination for each classifier. Fig 5 lists this subset of features for each classifier. The significance of each feature, as illustrated in Fig 5, reflects its frequency of selection across these classifiers.

A score of 5 on the figure indicates that the feature was selected by all five classifiers in their final feature subsets. Female Age was identified as a critical feature by all five classifiers, achieving the highest possible score of 5. Similarly, features such as AMH, endometrial thickness, number of oocytes, FSH, and number of embryo were selected by the majority of classifiers, with scores ranging from 4 to 5. In contrast, features like Vitamin D, Previous Pregnancy, and Cause of Infertility received lower scores, suggesting they were less frequently included in the feature subsets generated by the GA-based approach.

Also, ten features were identified as the most common ones: female age, AMH, Endometrial thickness, follicle size, number of oocytes, quality of retrieved oocytes (MI), quality of retrieved oocytes (MII), sperm count, sperm morphology, and quality of embryo.

Table 7 provides a detailed overview of features in the subset of a given classifier. Notably, RPART exhibits the highest number of 20 features in its subset, while SVM maintains the lowest number of 10 features.

**Table 7:** The selected features of dataset by different classification techniques.

classifier	Female age	AMH	AFC	FSH	LH	No. Transferred embryos	No embryos	Endometrial thickness	Sperm count	Sperm motility	Sperm morphology	Cause of infertility	Follicle size	No of retrieved oocytes	Quality of retrieved oocytes (MI)	Quality of retrieved oocytes (MII)	Quality of embryo	Duration of infertility	Male age	Previous pregnancy	Previous live birth	Vitamin D3	Total
ANN	√	√	√	√		√	√	√	√	√	√		√	√	√	√	√	√		√	√	√	19
RPART	√	√	√	√	√	√	√	√	√	√	√	√	√	√	√	√	√	√	√			√	20
RF	√	√		√		√	√	√	√		√		√	√	√	√	√		√				14
SVM	√	√						√	√		√		√	√	√	√	√						10
Adaboost	√	√		√			√	√	√		√		√	√	√	√	√						12
Total	5	5	2	4	1	3	4	5	5	2	5	1	5	5	5	5	5	2	2	1	1	2	

## 4. Discussion

This study attempted to utilize artificial intelligence in the IVF treatment practice through proposing a model to predict pregnancy outcome for IVF patients. For this, five well-known classifiers namely, ANN, RPART, RF, SVM and, AdaBoost were compared to select the most robust one, using the genetic algorithm as the feature selection method. A dataset of 812 records with 22 variables was used in this study.

The results demonstrate that SMOTE consistently improves classifier accuracy across all models. SMOTE is beneficial to data mining as it effectively addresses class imbalance issues by oversampling the minority class through the generation of synthetic instances. By creating new samples rather than simply duplicating existing ones, SMOTE helps mitigate overfitting, preserve the underlying data structure, and improve model generalization on unseen data [30]. These characteristics make SMOTE a valuable tool for enhancing model performance and accuracy in machine learning tasks, as it provides a more balanced dataset that can be easily integrated with various algorithms. Overall, SMOTE plays a crucial role in improving the robustness and reliability of machine learning models when dealing with imbalanced datasets [31, 32].

Our findings are in contrast with the results of a study, Hafiz et al. [33] did in 2017. They compared AdaBoost with four classifiers (SVM, RPART, RF and ANN) in which RF surpassed the others with an accuracy of 83.96 and AUC of 93.74. Contrary to our results, AdaBoost gained a relatively weak performance with an accuracy of 66.99 and an AUC of 47.52. This discrepancy could be due to the difference in model design between the two studies. The difference in the number of dataset records and the features used in the two studies can affect the model performance. Moreover, AdaBoost’s capacity to provide weights to each classifier’s predictions adaptively, improves the overall model’s robustness and efficacy. In this study, we constructed AdaBoost using the four other classifiers, i.e., SVM, RPART, RF, and ANN. This could also explain the significant difference between the performance of AdaBoost in the two studies.

Although little research has been done to compare Adaboost with other classifiers to develop an IVF outcome prediction model, but in many studies, RF has been compared to determine the highest performance. Their findings show that, in most of the cases, RF has surpassed other classifiers compared with [13, 22, 27, 34, 35]. This superiority of RF, AdaBoost and other similar methods may be due to their structure and how they predict. Known as ensemble learning methods, they are based on the idea of combining several different prediction models to create a global composite model that generates reliable and accurate estimates or predictions [36]. Ensemble learning methods seek to overcome overfitting by combining several ‘‘weak” models to form a diverse and accurate model. Theoretical and experimental evidence have shown that ensemble models achieve much better prediction performance than single models [37].

Another part of the findings depicts that applying Genetic algorithm as a feature selection method resulted in a relatively significant enhancement of the performance of all classifiers for each performance measure. Feature selection has a crucial role in eliminating irrelevant or redundant features, which can affect the model’s performance in predicting the IVF outcome accurately. Feature selection helps reduce the dimensionality of the dataset by selecting a subset of relevant features [38]. Genetic Algorithm, as an optimization approach based on the concept of biological evolution, has recently been more developed compared with different feature selection algorithms, and its advantages have been verified in various medical disciplines [39–41]. For the IVF prediction model, Guh et al. pointed out that GA performed better in attribute selection than other compared methods [42].

The results from Fig 5 demonstrate the robustness of GA in identifying key features for predicting IVF success. A score of 5 in the graph signifies that all five classifiers found a particular feature important when GA was employed for feature selection. ’Female Age’ emerged as the most consistently selected feature, aligning with clinical knowledge about its significant impact on fertility outcomes [43, 44]. This finding validates the GA’s ability to prioritize features crucial for prediction across multiple machine learning models [45]. Other important features, such as AMH, endometrial thickness, number of oocytes, and embryo quality, also achieved high selection scores, indicating their relevance across various classifiers. These features are well-known indicators of ovarian reserve, embryo viability, and overall reproductive potential, further supporting their inclusion in the models. On the other hand, features like Vitamin D, Previous Pregnancy, and Cause of Infertility were less frequently selected, which might suggest that their predictive value is either limited or highly context-dependent. This variation in selection across classifiers highlights the GA’s flexibility in evaluating complex interactions between features, which may be overlooked by simpler, filter-based methods.

While every classifier applied a different set of features in the prediction model, ten features were used in common by all of the five classifiers. Women’s age, AMH, Endometrial thickness, sperm count, sperm morphology, follicle size, retrieved oocytes, quality of retrieved oocytes (MI), quality of retrieved oocytes (MII) and quality of embryo are found to be the most important features affecting the IVF outcome. These features have been indicated to have a significant contribution to the prediction of IVF outcome in previous studies [27, 29, 46–48]. Selecting an appropriate feature subset is quite vital, as it can affect the prediction model notably. Curchoe et al. pointed out that, no precise set of features has been identified as the most important predictors in successful infertility treatment [49]. Although, the main objective of using feature selection methods is to determine the most suitable subset of significant predictors.

## 5. Strengths and limitation

This study highlights the ability of the capability of the predicting modelling using the Adaboost as an ensemble approach, combining the predictive performance of the four classifiers (ANN, RPART, RF, and SVM). This approach produces an ensemble model with reasonably high predicted accuracy for successful IVF by combining the mentioned classifiers. Furthermore, the research shows a significant boost in predicting accuracy by combining machine learning techniques with the Genetic Algorithm for feature selection. This novel approach improves the predictability of IVF success, giving significant insights for professionals and patients alike.

As the first limitation for this study, the findings are based on data from a single medical facility in Tehran, Iran. While the size of the data is reasonable, its applicability to different demographics and healthcare settings may be limited.

Finally, the prediction models created in this work should be externally validated in other studies to check the reliability and generalizability.

## 6. Conclusion

In this study, we presented a comparative study of machine learning methods integrated with Genetic Algorithm (GA) for predicting the success of In vitro fertilization (IVF) procedures. The study explores the performance of five machine learning algorithms: Artificial Neural Network (ANN), Recursive Partitioning and Regression Trees (RPART), Random Forest (RF), Support Vector Machine (SVM), and AdaBoost, using GA for feature selection.

The standout performer was Adaboost, achieving an accuracy of 89.8% with GA. Random Forest also showed promise, reaching 87.4% accuracy with GA. These results emphasize the significance of feature selection in improving model performance. Overall, the study highlights the effectiveness of ensemble learning methods like AdaBoost and Random Forest in predicting IVF outcomes.

Furthermore, the application of Genetic Algorithm as a feature selection method significantly improves the performance of all classifiers, emphasizing the importance of selecting relevant features for accurate IVF outcome prediction. Ten key features, including female age, AMH, endometrial thickness, sperm count, sperm morphology, follicle size, retrieved oocytes, quality of oocyte and embryo quality, are identified as critical factors influencing IVF success.

These models provide a promising tool for IVF practitioners, allowing for more exact treatment planning. We try to provide a better insight into the IVF success for infertile couples by applying AI and ML, which may potentially decrease emotional and financial difficulties.
